# Infections with Carbapenem-Resistant Gram-Negative Bacteria are a Serious Problem Among Critically Ill Children: A Single-Centre Retrospective Study

**DOI:** 10.3390/pathogens8020069

**Published:** 2019-05-21

**Authors:** Fatih Aygun, Fatma Deniz Aygun, Fatih Varol, Cansu Durak, Haluk Çokuğraş, Yıldız Camcıoğlu, Halit Çam

**Affiliations:** 1 Department of Pediatric Intensive Care Unit, Istanbul University Cerrahpasa Medical Faculty, Fatih, Istanbul 34098, Turkey; dr_fvarol@yahoo.com (F.V.); bzmrt@hotmail.com (C.D.); hacam@istanbul.edu.tr (H.Ç.); 2 Department of Infectious Disease, Istanbul University Cerrahpasa Medical Faculty, Fatih, Istanbul 34098, Turkey; fdenizaygun@gmail.com (F.D.A.); cokugras@gmail.com (H.Ç.); camciy@yahoo.com (Y.C.)

**Keywords:** carbapenem resistance, paediatric patients, nosocomial infection, colistin, tigecycline

## Abstract

Children in paediatric intensive care units (PICUs) are vulnerable to infections because invasive devices are frequently used during their admission. We aimed to determine the prevalence, associated factors, and prognosis of infections in our PICU. This retrospective study evaluated culture results from 477 paediatric patients who were treated in the PICU between January 2014 and March 2019. Ninety patients (18.9%) had bacterial infections, with gram-negative bacteria being the predominant infectious agents. Culture-positive patients were younger than culture-negative patients, and age was related to mortality and various clinical factors. Culture-positive bacterial infections in the PICU were associated with increased use of invasive mechanical ventilation (odds ratio(OR); 2.254), red blood cell (RBC) transfusions (OR:2.624), and inotropic drugs (OR:2.262). Carbapenem resistance was found in approximately one-third of gram-negative bacteria, and was most common in tracheal aspirate specimens and cases involving *Klebsiella* spp. Total parenteral nutrition was a significant risk factor (OR:5.870). Positive blood culture results were associated with poorer patient survival than other culture results. These findings indicate that infections, especially those involving carbapenem-resistant bacteria, are an important issue when treating critically ill children.

## 1. Introduction

Infections are a common cause of mortality in the paediatric intensive care unit (PICU) [1,2,3], with mortality rates of up to 50% depending on the infection’s origin [1]. Infants and young children have especially high risks, based on the immaturity of their immune system, their lack of prior exposure to antigens, and their physical barriers’ relative permeability to microorganisms [3]. In addition, children in the PICU are vulnerable to infections because invasive devices are frequently used during their hospitalization [2]. Therefore, the incidence of nosocomial infections in the PICU is higher than in other wards [4]. Nosocomial infections are becoming increasingly common in intensive care units, with higher rates of carbapenem-resistant bacterial infections (CRI), which has led to increased use of colistin and other last resort antibiotics [5]. In addition, MDR infections in children, particularly CRI in the PICU, are poorly described in the literature [6]. Therefore, early infection detection is critical in the PICU, and we aimed to determine the prevalence, risk factors, and prognosis of infections in our PICU.

## 2. Results

### 2.1. Demographic Characteristics

**Figure 1:**
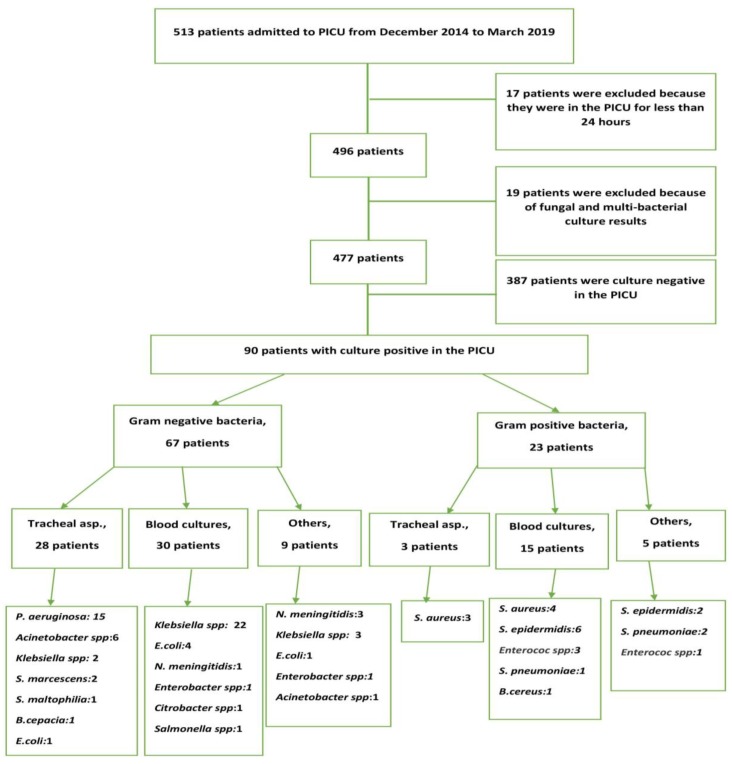
Study flow chart.

Between January 2014 and March 2019, 513 children were admitted to our PICU and 477 patients were considered eligible for the present study (Figure 1). Thirty-six patients were excluded because of a PICU stay duration of <24 h or death within the first 24 h after hospitalization (17 cases) or fungal and multi-bacterial culture results (19 cases).

The demographic characteristics of the 477 included patients are shown in Table 1. The patients included 265 males (55.6%) and 212 females (44.4%), with age ranging from 1 day to 18 years (median age: 2.0 years). The most frequent causes of PICU admission were respiratory disorders (188 patients; 24.7%), metabolic diseases (85 patients; 17.8%), sepsis (61 patients; 12.8%), surgery (57 patients; 11.9%), and neurological diseases (50 patients; 10.5%). The median PICU stay duration was 3.0 days (range: 2–122 days). Inotropic drugs were used for 103 patients (21.6%). Invasive mechanical ventilation (IMV) was used for 194 patients (40.7%) and non-invasive mechanical ventilation (NIV) was used for 161 patients (33.8%). Continuous renal replacement therapy (CRRT) was performed for 100 patients (21.0%). Thirty-seven patients (7.8%) died during their PICU stay. The central catheter-related bloodstream infection rate was 2.12/1000 catheter-days and the ventilator-associated pneumonia rate was 4.98/1000 ventilator-days.

### 2.2. Factors Associated with Culture-Positive Bacterial Infections in the PICU

There were relationships between culture-positive bacterial infections and patient age, IMV support, duration of IMV use, inotropic drug use, acute kidney injury (AKI), NIV support, mortality, duration of NIV use, duration of PICU stay, body temperature at admission, intravenous immunoglobulin usage, red blood cell (RBC) transfusion, and lactate concentration at admission Table 2 presents the comparison of prognostic factors in PICU according to culture-positive bacterial infections. This table was created considering that culture-positive bacterial infections may affect prognostic factors. In addition, we used the control group who were blood culture negative patients in this study.

Logistic regression analysis revealed that culture-positive bacterial infections in the PICU were significantly associated with IMV use (odds ratio [OR]: 2.254, 95% confidence interval [CI]: 1.229–4.131), RBC transfusions (OR: 2.624, 95% CI: 1.346–5.115), and inotropic drug use (OR: 2.262, 95% CI: 1.154–3.581) (Table 3).

### 2.3. Factors Associated with Gram-Positive and Gram-Negative Bacterial Infections in the PICU 

There was an association between gram-negative bacterial infections and IMV support (p = 0.045). There was also an association between gram-positive bacterial infections and catheter-related sepsis (p = 0.006) (Table 4).

### 2.4. Factors Associated with Gram-Negative CRIs 

There were associations between gram-negative CRIs and IMV support (p = 0.019), duration of PICU stay (p = 0.031), previous hospitalization (p = 0.045), RBC transfusion (p = 0.007), total parenteral nutrition use before infection (p < 0.001), culture results (p < 0.001), and culture sample source (p = 0.007). In the carbapenem-resistant bacterial infections, there were the most common *Klebsiella* spp (n = 9), *Pseudomonas aeruginosa* (n = 8), and *Acinetobacter* spp. (n = 6), respectively. Our CRIs were healthcare-associated and most of these patients had used total parenteral nutrition before the PICU admission. In the CRI infections, intravenous colistin was used with tigecycline, rifampin or nebulized colistin, after the antibiogram and culture results (Table 5).

Logistic regression analysis revealed that CRIs in the PICU were significantly associated with IMV use (OR: 7.626, 95% CI: 1.010–57.580), RBC transfusions (OR: 0.094, 95% CI: 0.017–0.525), and total parenteral nutrition use (OR: 5.870, 95% CI: 1.386–24.870) (Table 6).

### 2.5. Survival Outcomes According to Culture Results 

**Figure 2:**
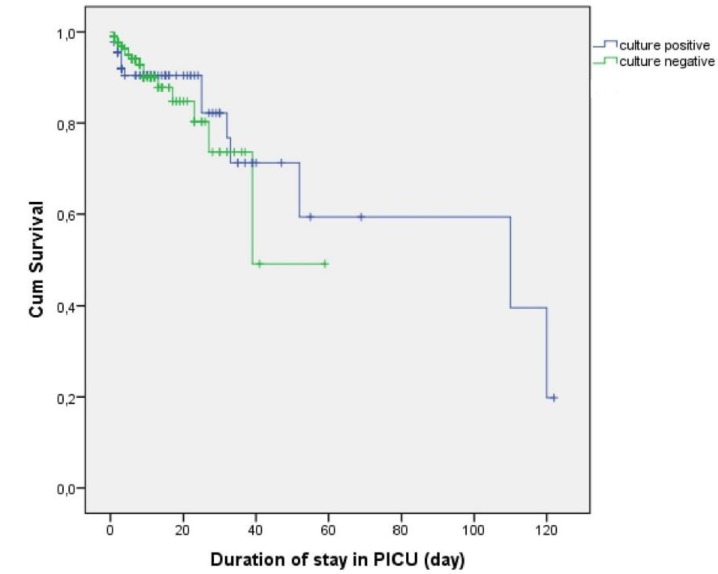
Kaplan–Meier curves for survival according to culture results.

Kaplan–Meier curves and the log-rank test revealed no significant difference in survival between patients with positive or negative culture results during their PICU stay (p = 0.945) (Figure 2).

**Figure 3:**
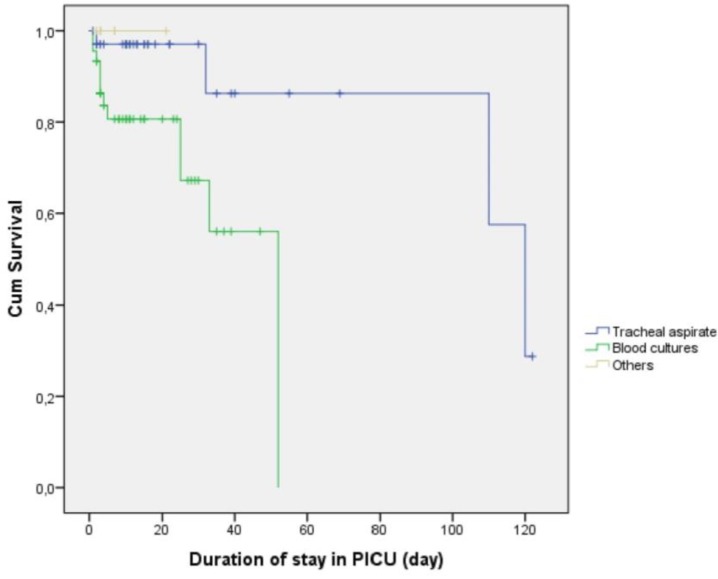
Kaplan–Meier curves for survival according to culture sample source.

However, a significant difference in survival was detected when we performed the analysis according to the culture sample source, with positive blood cultures being associated with the poorest survival, relative to the other sources (p = 0.012; Figure 3).

**Figure 4:**
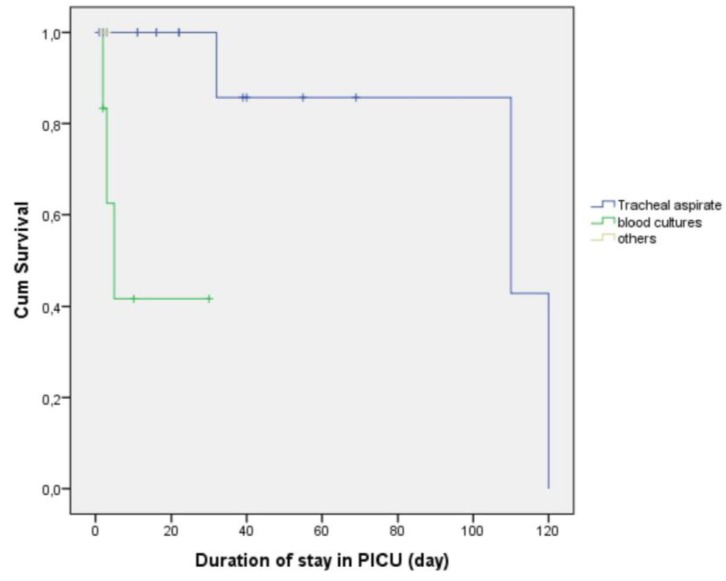
Kaplan–Meier curves for survival according to culture sample source in the CRIs patients (p = 0.004).

When we performed the analysis according to the culture sample source in the CRIs patients (n = 25), a significant difference in survival was detected. The positive blood cultures being associated with the poorest survival, relative to the other sources (p = 0.004; Figure 4).

## 3. Discussion

The present study revealed that infections in our PICU were associated with poor outcomes among critically ill children, which highlights the importance of understanding the causes of PICU infections. It highlights the importance of early detection and appropriate management. Among the 90 patients (18.9%) with bacterial infections, the infectious agents were predominantly gram-negative bacteria, although mortality was not associated with gram-negative or gram-positive status. However, patients with positive culture results were younger than patients with negative culture results, and age was associated with mortality and various clinical factors. Cases with CRIs were frequently detected using tracheal aspirate samples and frequently involved mechanical ventilator use, with the infectious agents typically being *Klebsiella* spp. and *Pseudomonas* spp. Our CRIs were healthcare-associated and most of these infected patients had used total parenteral nutrition before the PICU admission. Positive blood cultures were associated with poorer survival than positive tracheal aspirate cultures, and no deaths were observed in cases with isolated positive results from urine or cerebrospinal fluid cultures.

Most infections in our study involved gram-negative bacteria, and recent studies have focused on the treatment and prevention of gram-negative bacterial infections, given the rise in drug resistance [7,8,9,10]. Many mechanisms were described about the development of carbapenem resistance. Multidrug efflux pumps are very important in this setting [11,12,13]. This mechanism may be related to the increasing rates of CRIs, which can also involve multidrug resistance [14,15]. Recently, we observed that the frequency of CRIs increased in our PICU. Therefore, we have planned this study. Carbapenem resistance was identified in approximately one-third of gram-negative culture results, with 80% of these infections involving paediatric ward patients. The present study revealed CRIs in 25 patients, with 20 of these cases detected at the PICU admission. Most cases involved positive tracheal aspirate cultures and the most important risk factor for CRIs was previous hospitalization.

In 23 patients, we used tigecycline, rifampin, and nebulised colistin for salvage treatment in addition to IV colistin treatment. All these drugs, we used in various combinations with other antibiotics. CRIs have the potential to develop resistance during single-agent therapy. In the literature, tigecycline monotherapy used in the multidrug-resistant Gram-negative bacilli and tigecycline resistance had reported [16,17]. In a recent study showed that triple or double combination of meropenem, colistin and tigecycline were bactericidal against carbapenem-resistant *Klebsiella pneumoniae* isolates [18]. In another study, it was reported that combination antibiotic therapy including colistin and tigecycline may have better ability in preventing the evolution of resistance in CRIs [19]. However, another two studies reported that resistant strains were isolated during the combination therapy of colistin and tigecycline [20,21]. We think that combined colistin and tigecycline treatment provided synergistic effect against CRIs, like the first studies. The mortality of colistin-tigecycline combination treatment was limited to only 1 patient. However, there were 9 patients who used tigecycline-colistin combination treatment usage only. Rifampicin-based regimens have been shown to be effective against carbapenem-resistant *A. baumannii* infection. Because ıt was showed colistin-rifampicin combinations inhibit producing the bacterial biofilm [22]. Therefore, we used rifampicin all of the carbapenem-resistant *Acinetobacter spp.*

Intensive care units are the most common location for nosocomial infections because of invasive procedures [2,3,8,10]. In developed countries, invasive device-associated bloodstream infections and respiratory infections are the most common types of nosocomial infections in the PICU [3,10]. For example, ventilator-associated pneumonia and nosocomial infections are more common in patients with mechanical ventilation use [23,24]. The clearest risk factor for these infections is reportedly the duration of IMV usage [25]. In the present study, our nosocomial infection rate was low, although most infections were detected at admission and infections were predominantly detected using tracheal aspirate samples. Logistic regression analysis revealed that IMV use was associated with a 2.25-fold higher likelihood of infection, although prolonged IMV use was not associated with an increased likelihood of infection. 

Another risk factor for infections in the PICU is vascular catheter use [26], and it is important to identify and manage related risk factors, as catheter-related infections have high mortality and morbidity rates [27]. These infections most commonly involve gram-positive cocci (coagulase-negative staphylococci, such as *S. aureus*) [28]. However, the present study failed to detect significant differences in infection rates according to catheter type or location, and the logistic regression analysis revealed that catheter use was not associated with an increased infection rate. This may be related to our centre’s adoption of a “central line bundle” strategy for preventing infections, which was developed based on the existing literature [29]. The most common catheter-related bacterial infections involved gram-positive bacteria in our PICU, which agrees with previous findings [28]. However, a positive blood culture result for *S. epidermidis* does not necessarily confirm an infection, therefore we used other infection markers and clinical findings to confirm the same.

Blood component transfusions are also reportedly a risk factor for infection [30], and can increase the risk of mortality through immunosuppression and the increased risk of nosocomial infection [31]. The present study revealed that culture-positive cases had an increased need for RBC transfusions (2.6-fold more likely in culture-positive cases), although RBC transfusions were not associated with gram-negative or gram-positive culture results. Interestingly, greater RBC transfusion use was associated with a lower rate of CRIs. However, we could not explain this difference.

Among critically ill patients, AKI is an independent predictor of a poor prognosis [32]. This may be related to AKI being associated with an increased risk of infection, which is related to uremic retention and metabolic acidosis having negative effects on immunity [33]. Exposure to catheters and other invasive therapies, such as CRRT, may also increase the risk of infection. For example, the rates of infection and sepsis were higher in adult patients with AKI treated using CRRT than patients treated without CRRT [34]. Santiago et al. also recently reported a high infection rate among critically ill children treated using CRRT, especially in cases with >4 days of CRRT use [35]. The present study revealed that AKI was associated with a higher infection rate, although CRRT use was not associated with infection risk. Most patients who required CRRT had infections that were detected at admission, and the logistic regression analysis revealed no relationships between culture-positive infections and AKI or CRRT.

Akturk et al. recently reported that previous surgery was an independent risk factor for carbapenem-resistant *Klebsiella* infection in the PICU [10]. In contrast, the present study predominantly identified infections using tracheal aspirate samples, and previous surgery (92.0% of patients) was not associated with the risk of infection. Gram-negative CRIs were associated with a mortality rate of 24% and gram-negative CRIs did not have a significantly higher mortality rate. However, CRIs were associated with IMV use and prolonged hospitalization, with the logistic regression analysis revealing that CRIs were associated with a 7.626-fold higher likelihood of IMV use. The present study also revealed that CRIs were most commonly detected in cases with total parenteral nutrition use. This relationship may be related to many PICU patients who received total parenteral nutrition before their admission, had concomitant chronic diseases, and/or had a previous hospitalization. The most common patients with CRIs came from the metabolism, infection and gastroenterology wards. This situation may have been caused by frequent use of total parenteral nutrition in these wards.

Previous reports have discussed the relationship between inotropic drug use and prognosis, although the results are conflicting. For example, one study revealed that delaying inotropic treatment was associated with increased mortality [36], while another study revealed that inotropic drug use was associated with a poor prognosis and mortality [37]. Therefore, we evaluated the relevance of inotropic drug use in our PICU, and found that culture positivity was associated with a 2.2620-fold higher likelihood of inotropic drug use. 

Most of our patients with positive culture results at their PICU admission had gram-negative bacterial infections. Our CRIs were healthcare-associated and most of these patients had used antibiotics before the PICU admission. Therefore, the patients with positive culture results at admission had significantly longer PICU stays, because of longer treatment time. Nosocomial infections were also associated with prolonged PICU stays, which agrees with the findings of Dominguez et al. [38].

Bloodstream infections are a major cause of morbidity and mortality in critically ill patients. Our survival analysis revealed that positive blood culture results were associated with poorer survival than positive tracheal aspirate culture results. In a recent study showed that mortality of bloodstream infections was high and having resistant bacteria were independently associated with 30-day mortality [39]. Similarly, higher mortality was found in CRI related bloodstream infections in our study (Figure 4). Three of 6 patients with CRI related bloodstream infections died and all of them had *Klebsiella pneumoniae*. They died within 2, 3, and 5 days respectively. Another common point in these patients, they were admitted to the intensive care unit with the diagnosis of septic shock. The positive CRI in tracheal aspirate were 17 patients and three of them were died. In the tracheal aspirate cultures, two of these patients had *A. baumannii* and one had *Pseudomonas* spp. They were died in 32, 110 and 120 days respectively. Another common point in these three patients was that they had a previous history of intubation and PICU stay. In contrast, all patients with isolated positive results from urine or cerebral spinal fluid cultures survived and had relatively short PICU stays.

The present study’s findings are limited by the retrospective single-centre design, low number of patients, and heterogeneous populations (different ages). In addition, it is associated with risks of bias and the possibility that different results may be observed in other centres.

## 4. Materials and Methods

### 4.1. Study Design

PICU at Istanbul University-Cerrahpaşa has 7 beds, 7 ventilators, and 2 isolation rooms for treating children who are 1 day to 18 years old. For invasive procedures such as CRRT and extracorporeal membrane oxygenation (ECMO), newborns were rarely admitted to the PICU. Electronic and medical records were retrospectively searched to collect data regarding the 477 patients who were admitted to the PICU for various critical illnesses between January 2014 and March 2019. We excluded patients with a PICU stay duration of <24 h, patients who died during the first day after admission, and patients with fungal and multi-bacterial culture results. The study was conducted in accordance with the Declaration of Helsinki. The study’s retrospective protocol was approved by the institutional ethics committee (İstanbul University-Cerrahpaşa, 29430533-903.99-184025, 21 May 2018), and all study-related anonymized data are available upon reasonable request.

### 4.2. Data Collection and Patient Comparisons

The study variables included demographic and clinical factors, such as sex, age, invasive or non-invasive mechanical ventilation use, duration of PICU stay, mortality, Pediatric Risk of Mortality Score (PRISM) III score, CRRT use, haemogram findings, biochemistry results, and blood gases at admission. Patients were compared according to whether they had positive or negative culture findings, gram-negative or gram-positive infections, and infections that did or did not involve carbapenem resistance. 

We defined AKI as oliguria (urine output of <0.5 mL/kg/h) and an elevated serum creatinine value for the patient’s age or a 1.5-fold increase in the creatinine concentration at 24 h (relative to admission). The estimated glomerular filtration rate was calculated according to the original Schwartz formula, based on serum urea and creatinine concentrations that were measured using standard laboratory procedures.

The PRISM III score was calculated via a tool available on the Turkish Ministry of Health website (https://kalite.saglik.gov.tr/). This tool calculates the PRISM score based on the values for the lowest/highest systolic blood pressures from the first 24 h, diastolic blood pressure, heart rate, respiratory rate, PaO_2_/FiO_2_, PaCO_2_, prothrombin time/partial thromboplastin time, serum total bilirubin, calcium, potassium, glucose, bicarbonate, pupillary response, and Glasgow coma score.

### 4.3. Evaluation of Culture Results

A single blood culture was performed for each case to identify any known pathogens. In cases with a positive result, confirmation of infection was based on a second positive blood culture or detection of the same pathogen in separate peripheral and central blood cultures [2]. Tracheal aspirate samples were used to diagnose pneumonia in cases with a count of >10^3^ colony-forming units/mL [3]. Urine cultures were considered positive in cases with a count of >10^5^ colony-forming units/mL [4].

### 4.4. Statistical Analysis

All statistical analyses were performed using IBM SPSS software (version 21.0; IBM Corp., Armonk, NY, USA). Continuous data were expressed as median (range) and categorical data were expressed as number (percentage). Continuous variables were compared using Student’s t-test for parametric data and the Mann-Whitney U-test for non-parametric data. Categorical variables were compared using the chi-squared test or Fisher’s exact test. Univariate binary logistic regression models were used to calculate the relationships between risk factors and CRIs infections, culture positive infections with the results reported as ORs and 95% CIs. Survival data were calculated using Kaplan–Meier Log-Rank statistical analysis for cultures results and culture sample source. Differences were considered statistically significant at p-values of <0.05. 

## 5. Conclusions

The present study revealed that infections, especially CRIs, were associated with poor outcomes among critically ill children in the PICU. In addition, infections in the PICU were associated with increased use of IMV, RBC transfusions, and inotropic drugs. Furthermore, carbapenem resistance was found in approximately one-third of gram-negative culture results, and total parenteral nutrition was a risk factor for developing CRIs. We conclude that CRIs are a serious issue among critically ill children, and that reducing the inappropriate use of antibiotics, especially carbapenems, will be important in preventing multidrug-resistant bacterial infections.

## Figures and Tables

**Table 1:** Demographic characteristics of the 477 patients in the paediatric intensive care unit between January 2014 and March 2019.

Sex	
Male	265 (55.6%)
Female	212 (44.4%)
**Reason for Hospitalisation**	
Respiratory disease	118 (24.7%)
Metabolic disease	85 (17.8%)
Sepsis	61 (12.8%)
Surgery	57 (11.9%)
Neurologic disease	50 (10.5%)
Haematology-oncology	30 (6.3%)
Nephrology	28 (5.9%)
Cardiovascular disease	16 (3.4%)
Other	32 (7.2%)
Age	2.0 years (1 day to 18 years)
Acute kidney injury	137 (28.7%)
Inotropic medication	103 (21.6%)
Continuous renal replacement therapy	100 (21.0%)
Invasive mechanical ventilation	194 (40.7%)
Duration of PICU stay (days)	3.00 (2–122)
Central venous catheter	251 (52.6%)
Red blood cell transfusion	216 (45.3%)
Non-invasive mechanical ventilation	161 (33.8%)
Mortality	37 (7.8%)
Intravenous immunoglobulin use	55 (11.5%)
Infections detected at PICU admission	71 (14.9%)
Nosocomial infection in the PICU	19 (4.0%)
Total parenteral nutrition use	126 (26.4%)
Previous hospitalization	250 (52.4%)

Values are reported as number (percentage) or median (range). PICU, paediatric intensive care unit.

**Table 2:** Factors associated with culture-positive bacterial infections.

	Culture Positive (n = 90)	Culture Negative (n = 387)	P
Sex			
Male	49 (54.4%)	216 (55.8%)	0.814
Female	41 (45.6%)	171 (44.2%)	
Age (years)	1.0 (1 month–18 years)	2.25 (1 day–18 years)	0.013
IMV support	58 (64.4%)	136 (35.1%)	<0.001
Duration of IMV support (days)	10 (1–110)	3 (1–50)	<0.001
Inotropic drug use	39 (43.3%)	64 (16.5%)	<0.001
Acute kidney injury	50 (55.6%)	87 (22.5%)	<0.001
Continuous renal replacement therapy	31 (34.4%)	69 (17.8%)	0.077
NIV	51 (56.7%)	110 (28.4%)	<0.001
Duration of NIV support (h)	8 (1–144)	3 (1–120)	0.004
Mortality	13 (14.4%)	24 (6.2%)	0.008
Duration of PICU stay (days)	10 (1–122)	3 (1–59)	<0.001
Surgery	14 (15.6%)	49 (12.7%)	0.436
Body temperature at admission (°C)	37.0 (34.5–39.3)	36.7 (33.9–39.4)	0.002
Intravenous immunoglobulin use	30 (33.3%)	25 (6.5%)	<0.001
Red blood cell transfusion	60 (66.7%)	156 (40.3%)	<0.001
C-reactive protein (mg/dL)	2.0 (0.3–32.2)	1.0 (0.1–279.3)	0.244
Leucocyte count (/mm^3^)	11,800 (200–111,300)	10,800 (100–192,000)	0.206
Neutrophil count (/mm^3^)	6400 (0–105,200)	6150 (0–54,400)	0.089
Lactate (mmol/L)	1.75 (0.5–17.0)	1.70 (0.3–20.0)	0.018
Total parenteral nutrition use	40 (44.4%)	86 (22.2%)	<0.001

Values are reported as number (percentage) or median (range). PICU, paediatric intensive care unit; IMV, invasive mechanical ventilation, NIV, non-invasive mechanical ventilation.

**Table 3:** Logistic regression analysis of prognostic factors for culture-positive bacterial infections.

	P	Odds Ratio	95% Confidence Interval
Invasive mechanical ventilation	0.009	2.254	1.229–4.131
Catheter use	0.328	1.419	0.703–2.866
Surgery	0.497	1.270	0.637–2.530
Red blood cell transfusion	0.005	2.624	1.346–5.115
Acute kidney injury	0.478	1.312	0.620–2.777
Previous hospitalization	0.180	1.475	0.836–2.602
Inotropic drug use	0.017	2.262	1.154–4.433
Mortality	0.561	1.339	0.500–3.581

**Table 4:** Factors associated with gram-positive and gram-negative bacterial infections.

	Gram-Negative (n = 67)	Gram-Positive (n = 23)	P
Sex			
Male	34 (54.4%)	14 (55.8%)	0.401
Female	33 (45.6%)	9 (44.2%)	
Age (years)	1.0 (1 month–18 years)	1.80 (1 month–17 years)	0.139
Invasive mechanical ventilation support	50 (64.4%)	12 (35.1%)	0.045
Inotropic drug use	35 (43.3%)	10 (16.5%)	0.468
Acute kidney injury	21 (55.6%)	8 (22.5%)	0.833
Continuous renal replacement therapy	14 (34.4%)	5 (17.8%)	0.932
Non-invasive mechanical ventilation	31 (56.7%)	16 (28.4%)	0.054
Mortality	14 (14.4%)	2 (6.2%)	0.187
Duration of PICU stay (days)	9 (1–120)	12 (3–122)	0.665
Body temperature at admission (°C)	37.0 (34.5–39.3)	37.2 (35.2–38.7)	0.588
Intravenous immunoglobulin use	23 (33.3%)	10 (6.5%)	0.598
Catheter-related sepsis	3 (4.5%)	5 (17.8%)	0.006
Red blood cell transfusion	48 (66.7%)	15 (40.3%)	0.261
C-reactive protein (mg/dL)	2.0 (0.3–32.2)	2.0 (0.4–30.6)	0.433
Leucocyte count (/mm^3^)	13,600 (200–111,800)	10,300 (200–40,500)	0.194
Neutrophil count (/mm^3^)	7200 (0–105,200)	5800 (100–39,500)	0.333

Values are reported as number (percentage) or median (range). PICU, paediatric intensive care unit.

**Table 5:** Factors associated with gram-negative carbapenem-resistant bacterial infections.

		Carbapenem-Resistant Gram-Negative		P
		Yes (n = 25)	No (n = 42)	
Sex	Female	10 (40.0%)	25 (59.5%)	0.122
	Male	15 (60.0%)	17 (40.5%)	
Age (years)		1.0 (2 month–18 years)	1.0 (1 month–17 years)	0.289
Duration of IMV support (days)		23 (92.0%)	28 (66.7%)	0.019
Inotropic drug use		9.0 (1–110)	9.5 (1–52)	0.559
Inotropic drug use		10 (40.0%)	25 (59.5%)	0.122
Acute kidney injury		6 (24.0%)	15 (35.7%)	0.320
Continuous renal replacement therapy		3 (12.0%)	10 (23.8%)	0.237
NIV		11 (44.0%)	20 (47.6%)	0.774
Duration of NIV support (h)		48.0 (2–144)	6.0 (1–96)	0.083
Mortality		6 (24.0%)	7 (16.7%)	0.463
Duration of PICU stay (days)		11.0 (1–120)	7.5 (1–52)	0.031
Intravenous immunoglobulin use		6 (24.0%)	17 (40.5%)	0.275
Previous hospitalization		23 (92.0%)	30 (71.4%)	0.045
Surgery		3 (12.0%)	3 (7.1%)	0.501
Red blood cell transfusion		12 (48.0%)	35 (83.3%)	0.007
Central venous catheter use		19 (76.0%)	33 (78.6%)	0.807
Total parenteral nutrition usage before infection		19 (76.0%)	15 (35.7%)	<0.001
Positive culture samples taken at admission		20 (80.0%)	36 (85.7%)	0.541
Culture results	*Klebsiella* spp.	9 (36.0%)	18 (42.9%)	<0.001
	*P. aeruginosa*	8 (32.0%)	7 (16.7%)	
	*Acinetobacter* spp.	6 (24.0%)	1 (2.4%)	
	Others	2 (8.0%)	16 (38.1%)	
Culture sample source: Antibiotics used for salvage treatment in addition to IV colistin treatment	Tracheal aspirate	17 (68.0%)	12 (28.6%)	0.007
	Blood	6 (24.0%)	22 (52.4%)	
	Others	2 (8.0%)	8 (19.0%)	
	Tigecycline	9 (36.0%)	-	
	Rifampin	11 (44.0)	-	
	Nebulised colistin	23 (92.0%)	-	

Values are reported as number (percentage) or median (range). PICU, paediatric intensive care unit; IMV, invasive mechanical ventilation, NIV, non-invasive mechanical ventilation.

**Table 6:** Prognostic factors for carbapenem-resistant bacterial infections.

	P	Odds Ratio	95% Confidence Interval
Invasive mechanical ventilation	0.005	7.626	1.010–57.580
Surgery	0.896	1.112	0.226–5.468
Red blood cell transfusion	0.007	0.094	0.017–0.525
Acute kidney injury	0.318	0.415	0.074–2.330
Previous hospitalization	0.560	1.965	0.203–19.046
Inotropic drug use	0.669	1.322	0.923–4.421
Mortality	0.706	1.341	0.292–6.156
Total parenteral nutrition use	0.016	5.870	1.386–24.870
